# Self-reported Daily, Weekly, and Monthly Cannabis Use Among Women Before and During Pregnancy

**DOI:** 10.1001/jamanetworkopen.2019.6471

**Published:** 2019-07-19

**Authors:** Kelly C. Young-Wolff, Varada Sarovar, Lue-Yen Tucker, Amy Conway, Stacey Alexeeff, Constance Weisner, Mary Anne Armstrong, Nancy Goler

**Affiliations:** 1 Division of Research, Kaiser Permanente Northern California, Oakland; 2 Department of Psychiatry, University of California, San Francisco; 3 Early Start Program, Kaiser Permanente Northern California, Oakland; 4 Regional Offices, Kaiser Permanente Northern California, Oakland

## Abstract

**Importance:**

As the overall prevalence of prenatal cannabis use rises, it is vital to also monitor trends in the frequency of cannabis use in the period leading up to and during pregnancy because more frequent use may confer greater health risks for mothers and their children.

**Objective:**

To examine trends in the frequency of self-reported cannabis use among pregnant women in the year before and during pregnancy.

**Design, Setting, and Participants:**

Cross-sectional study using data from 367 403 pregnancies among 276 991 women 11 years or older who completed a self-administered questionnaire on cannabis use during standard prenatal care in Kaiser Permanente Northern California from January 1, 2009, to December 31, 2017. The annual prevalence of self-reported daily, weekly, and monthly cannabis use among women before and during pregnancy was estimated using Poisson regression with a log link function, adjusting for sociodemographics. Data analyses were conducted from February to May 2019.

**Exposures:**

Calendar year.

**Main Outcomes and Measures:**

Self-reported frequency of cannabis use in the year before pregnancy and during pregnancy assessed as part of standard prenatal care (at approximately 8 weeks’ gestation).

**Results:**

Among the overall sample of 367 403 pregnancies among 276 991 women, 35.9% of the women self-reported white race/ethnicity; 28.0%, Hispanic; 16.6%, Asian; 6.0%, African American; and 13.5%, other. In the sample, 1.2% of the women were aged 11 to 17 years; 15.3%, 18 to 24 years; 61.4%, 25 to 34 years; and 22.0%, older than 34 years. Median (interquartile range) neighborhood household income was $70 472 ($51 583-$92 643). From 2009 to 2017, the adjusted prevalence of cannabis use in the year before pregnancy increased from 6.80% (95% CI, 6.42%-7.18%) to 12.50% (95% CI, 12.01%-12.99%), and the adjusted prevalence of cannabis use during pregnancy increased from 1.95% (95% CI, 1.78%-2.13%) to 3.38% (95% CI, 3.15%-3.60%). Annual relative rates of change in self-reported daily cannabis use (1.115; 95% CI, 1.103-1.128), weekly cannabis use (1.083; 95% CI, 1.071-1.095), and monthly or less cannabis use (1.050; 95% CI, 1.043-1.057) in the year before pregnancy increased significantly, with daily use increasing most rapidly (from 1.17% to 3.05%). Similarly, annual relative rates of change in self-reported daily cannabis use (1.110; 95% CI, 1.089-1.132), weekly cannabis use (1.075; 95% CI, 1.059-1.092) and monthly or less cannabis use (1.044; 95% CI, 1.032-1.057) during pregnancy increased significantly from 2009 to 2017, with daily use increasing most rapidly (from 0.28% to 0.69%).

**Conclusions and Relevance:**

Results of this study demonstrate that frequency of cannabis use in the year before pregnancy and during pregnancy has increased in recent years among pregnant women in Northern California, potentially associated with increasing acceptance of cannabis use and decreasing perceptions of cannabis-associated harms.

## Key Points

**Question:**

Has the frequency of cannabis use among pregnant women in the year before and during pregnancy increased in recent years?

**Findings:**

In this serial cross-sectional study of 367 403 pregnancies among women in Kaiser Permanente Northern California who were universally screened for self-reported cannabis use as part of standard prenatal care, annual relative rates of daily, weekly, and monthly cannabis use in the year before pregnancy and during pregnancy increased from 2009 to 2017. Relative rates of self-reported daily cannabis use in the year before and during pregnancy increased fastest.

**Meaning:**

Results of this study demonstrate that frequency of cannabis use in the year before pregnancy and during pregnancy has increased among women in Northern California in recent years, with relative rates of daily cannabis use increasing most rapidly.

## Teaser

This cross-sectional study of pregnancies among women in the Kaiser Permanente Northern California health care system examines trends in daily, weekly, and monthly self-reported cannabis use in the year before and during pregnancy from 2009 to 2017.

## Introduction

Cannabis use during pregnancy may adversely affect the health of pregnant mothers and the developing fetus. A growing body of literature suggests that prenatal cannabis use is associated with lower offspring birthweight, and there is evidence of possible adverse effects on other fetal and neonatal outcomes, as well as worse neuropsychological functioning among children exposed to cannabis in utero.^1,2,3,4,5^ While much is still unknown, national guidelines recommend that pregnant women abstain from cannabis use in the perinatal period owing to concerns about the negative health outcomes of cannabis use in pregnancy.^3^

Despite these recommendations, cannabis use among pregnant women has increased during recent years.^6,7^ This use has increased with general acceptance and accessibility of cannabis,^8,9^ and many women who are pregnant and cannabis users believe there is slight or no risk to using cannabis.^10,11^ Studies suggest that obstetric health care professionals often do not respond or provide counseling to women who disclose cannabis use during pregnancy,^12^ and women report dissatisfaction with the quality of information about the harms of prenatal cannabis use.^10,13^ This leaves patients to turn to online resources, social media, friends, and family where they frequently receive incomplete information suggesting that cannabis use in pregnancy is safe and effective in treating pregnancy-related symptoms.^13,14,15,16^

As the prevalence of prenatal cannabis use rises, research is needed to determine whether pregnant women are also using cannabis more frequently, as daily and weekly prenatal use may carry greater health risks than less frequent use.^17^ Available data indicate that the prevalence of daily or near-daily cannabis use among US adults increased from 1.9% in 2002 to 3.5% in 2014, while the prevalence of daily or near daily cannabis use among US adult users of cannabis increased from 18.0% in 2002 to 26.3% in 2014, corresponding with decreases in perceived risks associated with cannabis use.^18^ Further, cross-sectional data from US women from 2007 to 2012 found that among pregnant and nonpregnant women, 4% and 8% reported cannabis use in the past month, and 7% and 6% reported cannabis use in the past 2 to 12 months, respectively.^11^ Among pregnant and nonpregnant women with past-year cannabis use, 16% and 13% reported almost-daily cannabis use, and 48% and 33% reported using at least twice a week in the past year, respectively. An alarming 18% of pregnant women with past-year cannabis use met the criteria for a cannabis use disorder.^11^

As cannabis use becomes more acceptable and accessible,^8,19^ women may use cannabis increasingly frequently in the time period leading up to and during pregnancy. Despite an overall increase in the prevalence of prenatal cannabis use, to our knowledge, trends in cannabis use frequency among pregnant women have not been previously explored. Understanding whether daily and weekly prenatal cannabis use has increased in recent years is critical, as more frequent cannabis use in pregnancy may confer greater adverse health outcomes. Data on the changing epidemiology of daily and weekly prenatal cannabis use will inform research studies that evaluate the harms associated with cannabis use in pregnancy and guide educational programs and clinical interventions aimed at reducing use in this priority population.

To address this issue, we conducted this study to examine trends in daily, weekly, and monthly or less self-reported cannabis use in the year before and during pregnancy from 2009 to 2017, among a diverse population of pregnant women in Kaiser Permanente Northern California (KPNC), a large health care system with universal screening for cannabis use as part of standard prenatal care.

## Methods

### Data Source and Study Population

Kaiser Permanente Northern California is an integrated health care system serving more than 4 million racially and sociodemographically diverse patients who are representative of the Northern California region^20,21,22^; KPNC has more than 600 obstetric physicians and nurse practitioners, more than 100 certified nurse-midwives, and more than 45 000 pregnancies annually. Standard prenatal care includes universal drug screening by self-report (via a self-administered questionnaire) and urine toxicology testing at the first prenatal encounter (at approximately 8 weeks’ gestation). The study sample comprised pregnant women who completed a self-administered questionnaire that assessed self-reported cannabis use during the year before pregnancy and during pregnancy as part of usual prenatal care from January 1, 2009, to December 31, 2017. The KPNC institutional review board approved and this study with waiver of consent. This study followed the Strengthening the Reporting of Observational Studies in Epidemiology (STROBE) reporting guideline.

### Measures

Patients self-reported frequency of cannabis use (none, monthly or less, weekly, daily) in the year before pregnancy and during pregnancy was assessed via a self-administered questionnaire at intake to prenatal care. Daily cannabis use refers to use at least once per day, weekly cannabis use refers to at least once per week (but less than daily), and monthly or less cannabis use refers to at least once during the time frame (but less than weekly); frequency categories were mutually exclusive. Age group, self-reported race/ethnicity, and median neighborhood household income were extracted from the electronic health record (EHR). Median neighborhood household income was geocoded based on members’ addresses and reflects members’ neighborhoods, not individual data.

### Statistical Analysis

We examined demographic differences in self-reported frequency of cannabis use in the year before and during pregnancy using χ^2^ tests. We modeled the adjusted prevalence of self-reported daily, weekly, and monthly or less cannabis use before and during pregnancy annually using Poisson regression with a log link function in SAS 9.4 (SAS Institute, Inc). We adjusted for race/ethnicity, age, and median neighborhood household income using the average covariate distributions across the study period. Women were allowed to contribute more than 1 pregnancy to the analysis. As we were estimating annual prevalence for the population, rather than estimating how an individual risk factor influenced the odds of substance use, the analysis was not adjusted for multiple pregnancies per woman. We modeled linear trends in cannabis use frequency by including a linear term for calendar year and estimated the annual relative rate of change with 95% CIs. We also tested for differences in linear trends by race/ethnicity and median neighborhood household income. *P* < .05 (2-sided) was considered statistically significant.

## Results

Of 369 665 pregnancies among women in KPNC 11 years and older from 2009 to 2017, 2262 missing responses for self-reported cannabis use were excluded (0.6%). The final study sample comprised 367 403 pregnancies among 276 991 women; 75 234 women (27.2%) had more than 1 pregnancy during the study period. Self-reported race/ethnicity among women in the sample was 35.9% white, 28.0% Hispanic, 16.6% Asian, 6.0% African American, and 13.5% other. In this sample, 1.2% of the women were aged 11 to 17 years; 15.3%, 18 to 24 years; 61.4%, 25 to 34 years; and 22.0%, older than 34 years. Median (interquartile range) neighborhood household income was $70 472 ($51 583-$92 643).

Across years, self-reported cannabis use in the year before and during pregnancy was reported in 9.0% and 2.5% of pregnancies, respectively; 6.6% self-reported use only during the year before pregnancy, while 0.1% self-reported only use during pregnancy, and 2.4% self-reported use both in the year before pregnancy and during pregnancy. The majority (96.0%) of those with self-reported cannabis use during pregnancy also self-reported cannabis use during the year before pregnancy.

Demographic characteristics by frequency of cannabis use are presented in Table 1. More frequent cannabis use during the year before pregnancy and during pregnancy was associated with younger age group, African American race/ethnicity, and lower median neighborhood household income.

**Table 1.** Demographic Characteristics of 367 403 Pregnancies by Frequency of Self-reported Cannabis Use

Characteristics	No. (%)^a^									
	Cannabis Use in Year Before Pregnancy					Cannabis Use During Pregnancy				
	None (n = 334 392)	Monthly or Less (n = 18 744)	Weekly (n = 7038)	Daily (n = 7229)	*P* Value for χ^2^	None (n = 358 077)	Monthly or Less (n = 5080)	Weekly (n = 2423)	Daily (n = 1823)	*P* Value for χ^2^
Age, y										
11-17	3146 (71.1)	696 (15.7)	295 (6.7)	287 (6.5)	<.001	3998 (90.4)	268 (6.1)	92 (2.1)	66 (1.5)	<.001
18-24	44 950 (79.8)	5490 (9.7)	2592 (4.6)	3310 (5.9)		52 489 (93.2)	1990 (3.5)	1032 (1.8)	831 (1.5)	
25-34	209 403 (92.8)	9859 (4.4)	3327 (1.5)	3074 (1.4)		221 578 (98.2)	2271 (1.0)	1049 (0.5)	765 (0.3)	
>34	76 893 (95.0)	2699 (3.3)	824 (1.0)	558 (0.7)		80 012 (98.8)	551 (0.7)	250 (0.3)	161 (0.2)	
Race/ethnicity										
Asian	59 522 (97.4)	1172 (1.9)	247 (0.4)	152 (0.3)	<.001	60 779 (99.5)	215 (0.4)	61 (0.1)	38 (0.1)	<.001
African American	17 257 (78.6)	2144 (9.8)	1084 (4.9)	1479 (6.7)		20 052 (91.3)	927 (4.2)	530 (2.4)	455 (2.1)	
Hispanic	93 030 (90.6)	5490 (5.3)	1998 (1.9)	2207 (2.2)		100 144 (97.5)	1497 (1.5)	596 (0.6)	488 (0.5)	
Other	45 822 (92.3)	2162 (4.4)	792 (1.6)	853 (1.7)		48 497 (97.7)	619 (1.3)	283 (0.6)	230 (0.5)	
White	118 761 (90.0)	7776 (5.9)	2917 (2.2)	2538 (1.9)		128 605 (97.4)	1822 (1.4)	953 (0.7)	612 (0.5)	
Median household income, $^b^										
<51 583	80 460 (87.9)	5738 (6.3)	2443 (2.7)	2908 (3.2)	<.001	87 950 (96.1)	1869 (2.0)	940 (1.0)	790 (0.9)	<.001
51 583 to <70 472	82 772 (90.3)	5040 (5.5)	1873 (2.0)	1937 (2.1)		89 205 (97.4)	1316 (1.4)	636 (0.7)	465 (0.5)	
70 472 to <92 643	84 340 (92.1)	4310 (4.7)	1531 (1.7)	1400 (1.5)		89 670 (97.9)	1073 (1.2)	493 (0.5)	345 (0.4)	
≥92 643	86 031 (93.7)	3605 (3.9)	1178 (1.3)	969 (1.1)		90 416 (98.5)	800 (0.9)	349 (0.4)	218 (0.2)	

^a^ Self-reported cannabis use in the year before and during pregnancy was assessed via a questionnaire as part of standard prenatal care (at approximately 8 weeks’ gestation).

^b^ Data on median household income was missing for 868 women.

**Figure 1.**
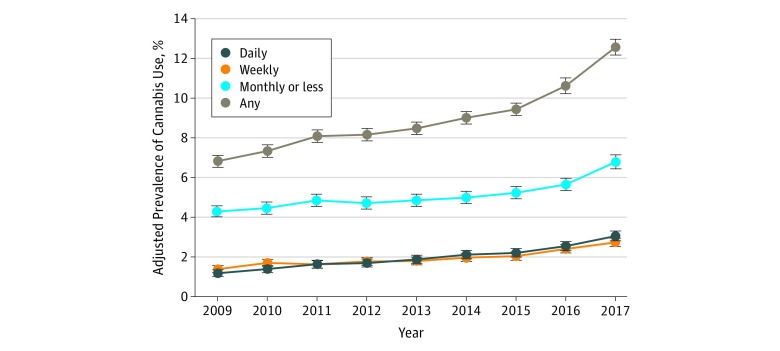
Adjusted Prevalence of Cannabis Use Among 367 403 Pregnancies in the Year Before Pregnancy by Frequency of Use, 2009-2017 Adjusted prevalence estimates (dots) and 95% CIs (error bars) were estimated from Poisson regression models controlling for age group, race/ethnicity, and median neighborhood household income (extracted from the electronic health record [Table 2]). Self-reported cannabis use in the year before pregnancy was assessed via a questionnaire as part of standard prenatal care (at approximately 8 weeks’ gestation).

From 2009 to 2017, the adjusted prevalence of self-reported cannabis use during the year before pregnancy increased significantly from 6.80% (95% CI, 6.42%-7.18%) to 12.50% (95% CI, 12.01%-12.99%). Daily use increased most rapidly at an annual relative rate of 1.115 (95% CI, 1.103-1.128), from 1.17% (95% CI, 1.04%-1.30%) to 3.05% (95% CI, 2.84%-3.26%) (Table 2; Figure 1). Weekly use increased at an annual relative rate of 1.083 (95% CI, 1.071-1.095), from 1.39% (95% CI, 1.26%-1.53%) to 2.73% (95% CI, 2.55%-2.91%), and monthly or less use increased at an annual relative rate of 1.050 (95% CI, 1.043-1.057), from 4.26% (95% CI, 4.00%-4.51%) to 6.74% (95% CI, 6.44%-7.05%). Among women who used cannabis during the year before pregnancy, the proportion of daily users increased from 17.1% (95% CI, 15.5%-18.7%) to 25.2% (95% CI, 23.7%-26.6%) and the proportion of weekly users increased from 20.4% (95% CI, 18.8%-22.0%) to 22.0% (95% CI, 20.8%-23.2%), while the proportion of monthly or less users decreased from 62.7% (95% CI, 60.5%-64.9%) to 53.1% (95% CI, 51.7%-54.5%).

**Table 2.** Adjusted Prevalence of Cannabis Use in Kaiser Permanente Northern California for Each Year and Annual Relative Rate of Change by Use Frequency Among 367 403 Pregnancies, 2009-2017

Cannabis Use Frequency^a^	Adjusted Prevalence of Cannabis Use Frequency, % (95% CI)^b^									Linear Trend Estimation	
	2009	2010	2011	2012	2013	2014	2015	2016	2017	Annual Relative Rate of Change Estimate (95% CI)	*P* Value
**Year Before Pregnancy**											
Any	6.80 (6.42-7.18)	7.33 (6.93-7.72)	8.07 (7.65-8.48)	8.16 (7.74-8.57)	8.47 (8.05-8.90)	9.00 (8.58-9.43)	9.42 (8.99-9.85)	10.59 (10.14-11.04)	12.50 (12.01-12.99)	1.071 (1.064-1.078)	<.001
Daily	1.17 (1.04-1.30)	1.38 (1.24-1.52)	1.63 (1.47-1.78)	1.68 (1.53-1.84)	1.87 (1.70-2.04)	2.11 (1.94-2.28)	2.20 (2.03-2.38)	2.54 (2.35-2.72)	3.05 (2.84-3.26)	1.115 (1.103-1.128)	<.001
Weekly	1.39 (1.26-1.53)	1.49 (1.35-1.63)	1.62 (1.47-1.76)	1.77 (1.61-1.92)	1.79 (1.64-1.95)	1.94 (1.79-2.10)	2.02 (1.86-2.18)	2.39 (2.22-2.56)	2.73 (2.55-2.91)	1.083 (1.071-1.095)	<.001
Monthly or less	4.26 (4.00-4.51)	4.46 (4.20-4.73)	4.83 (4.56-5.10)	4.70 (4.43-4.97)	4.81 (4.54-5.09)	4.95 (4.68-5.22)	5.21 (4.93-5.48)	5.68 (5.40-5.96)	6.74 (6.44-7.05)	1.050 (1.043-1.057)	<.001
**During Pregnancy**											
Any	1.95 (1.78-2.13)	2.07 (1.88-2.25)	2.37 (2.17-2.56)	2.30 (2.11-2.50)	2.38 (2.18-2.58)	2.61 (2.41-2.82)	2.74 (2.53-2.94)	2.97 (2.76-3.18)	3.38 (3.15-3.60)	1.065 (1.054-1.076)	<.001
Daily	0.28 (0.23-0.34)	0.36 (0.30-0.42)	0.45 (0.38-0.51)	0.38 (0.32-0.45)	0.45 (0.38-0.52)	0.57 (0.49-0.65)	0.58 (0.50-0.66)	0.68 (0.60-0.76)	0.69 (0.61-0.78)	1.110 (1.089-1.132)	<.001
Weekly	0.49 (0.42-0.56)	0.54 (0.47-0.61)	0.56 (0.49-0.64)	0.59 (0.52-0.67)	0.63 (0.55-0.70)	0.66 (0.58-0.74)	0.76 (0.68-0.84)	0.76 (0.68-0.84)	0.92 (0.83-1.01)	1.075 (1.059-1.092)	<.001
Monthly or less	1.18 (1.06-1.30)	1.17 (1.05-1.29)	1.36 (1.23-1.49)	1.33 (1.20-1.46)	1.30 (1.17-1.43)	1.39 (1.26-1.52)	1.39 (1.26-1.52)	1.53 (1.39-1.66)	1.77 (1.63-1.91)	1.044 (1.032-1.057)	<.001

^a^ Self-reported cannabis use in the year before and during pregnancy was assessed via a questionnaire as part of standard prenatal care (at approximately 8 weeks’ gestation).

^b^ Adjusted prevalence estimates and 95% CIs were estimated from Poisson regression models controlling for age group, race/ethnicity, and median neighborhood household income (extracted from the electronic health record).

**Figure 2.**
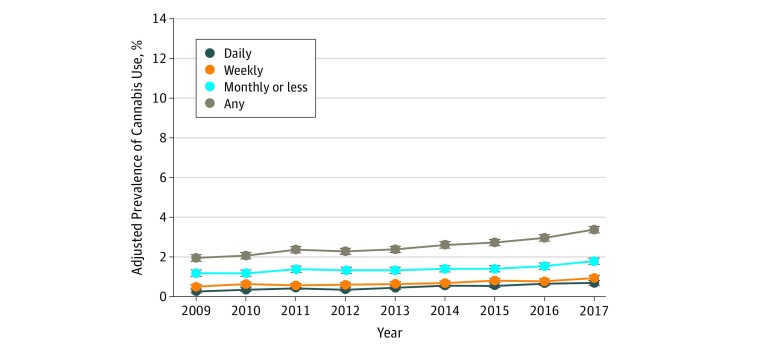
Adjusted Prevalence of Cannabis Use Among 367 403 Pregnancies During Pregnancy by Frequency of Use, 2009-2017 Adjusted prevalence estimates (dots) and 95% CIs (error bars) were estimated from Poisson regression models controlling for age group, race/ethnicity, and median neighborhood household income (extracted from the electronic health record [Table 2]). Self-reported cannabis use during pregnancy was assessed via a questionnaire as part of standard prenatal care (at approximately 8 weeks’ gestation).

The adjusted prevalence of self-reported cannabis use during pregnancy increased from 1.95% (95% CI, 1.78%-2.13%) in 2009 to 3.38% (95% CI, 3.15%-3.60%) in 2017. Daily use increased most rapidly at an annual relative rate of 1.110 (95% CI, 1.089-1.132), from 0.28% (95% CI, 0.23%-0.34%) to 0.69% (95% CI, 0.61%-0.78%) (Table 2; Figure 2). Weekly use increased at an annual relative rate of 1.075 (95% CI, 1.059-1.092), from 0.49% (95% CI, 0.42%-0.56%) to 0.92% (95% CI, 0.83%-1.01%), and monthly or less use increased at an annual relative rate of 1.044 (95% CI, 1.032-1.057), from 1.18% (95% CI, 1.06%-1.30%) to 1.77% (95% CI, 1.63%-1.91%). Among women who self-reported using cannabis during pregnancy, the proportion of daily users increased from 14.6% (95% CI, 12.0%-17.2%) to 20.9% (95% CI, 18.5%-23.3%) and the proportion of weekly users increased from 25.1% (95% CI, 22.1%-28.2%) to 27.4% (95% CI, 24.9%-29.8%), while the proportion of monthly or less users decreased from 60.3% (95% CI, 56.6%-64.1%) to 51.8% (95% CI, 49.2%-54.4%).

Annual relative rates of increases in daily, weekly and monthly cannabis use in the year before pregnancy and during pregnancy were consistent across racial/ethnic and household income groups. The only statistically significant difference we identified was a small difference by race for monthly or less cannabis use in the year before pregnancy, where the rate of increase varied from 1.02 (among African American women) to 1.07 (among Asian women) (*P* = .01).

## Discussion

We found that self-reported daily, weekly, and monthly cannabis use before and during pregnancy increased from 2009 to 2017 in a large sample of pregnant women universally screened for cannabis during standard prenatal care. Daily use increased most rapidly, reaching 25% among those who used during the year before pregnancy and 21% among those who used during pregnancy by 2017. The prevalence of cannabis use via urine toxicology testing has also increased significantly over time,^7^ suggesting that results are not simply due to greater willingness to self-report prenatal cannabis use in recent years.

Frequent cannabis use among pregnant women raises important public health concerns, as initial evidence suggests that heavier use might be associated with worse neonatal health outcomes.^17^ Despite this risk, however, US data suggest that 71% of pregnant women who used cannabis in the past year perceive no or slight risk in using cannabis once or twice a week.^11^ Pregnant women who use cannabis more frequently during pregnancy are also more likely to use other drugs,^17^ and future research is critically needed to examine the short- and long-term health outcomes for mothers and their offspring associated specifically with daily vs occasional cannabis use during different time points in pregnancy, adjusting for co-use of other substances.

Consistent with prior studies,^11,18,23^ we found a higher prevalence of any cannabis use and more frequent cannabis use in the year before and during pregnancy among women with lower median neighborhood household income, African American race/ethnicity, and younger age group. Additional research is needed to understand the mechanisms that underlie sociodemographic differences in the prevalence and frequency of cannabis use in pregnancy. However, annual relative rates of increases in daily, weekly, and monthly or less cannabis use in the year before pregnancy and during pregnancy were similar regardless of race/ethnicity and household income, suggesting that cannabis use frequency is rising consistently across these groups of pregnant women.

Pregnant women may use cannabis for therapeutic reasons or as a natural substitute for prescribed medications used to treat mental health and pregnancy-related symptoms.^10^ Women report using cannabis during pregnancy to manage mood, stress, and morning sickness,^10,23^ and prenatal cannabis use is elevated among women with depressive symptoms, poor mental health, and nausea and vomiting in pregnancy.^24,25,26,27,28,29^ Obstetric clinicians can play a key role in preventing harms associated with cannabis use in pregnancy by educating patients about the potential risks of frequent use, advising all patients who are pregnant to quit cannabis use, and providing patients with safe and effective medically approved ways to improve mood and treat nausea and vomiting in pregnancy. Notably, the high prevalence of cannabis use during the year before pregnancy among those who self-report use during pregnancy (96%) suggests that educational and prevention efforts geared to reduce prenatal use should begin for women of reproductive age before they become pregnant.

To date, 33 states and the District of Columbia have legalized medicinal cannabis use and 11 states and the District of Columbia have legalized recreational cannabis use.^30^ Public support for cannabis legalization is increasing, with 64% of US adults favoring cannabis legalization in 2018.^8^ This shift in support for cannabis legalization is driven largely by reproductive-aged adults aged 18 to 35 years.^8^ While the consequences of legalization on prenatal cannabis use are unclear, initial data from Maryland suggest that nearly two-thirds (62%) of cannabis users who are pregnant reported that they would increase their cannabis use in pregnancy if cannabis were legalized,^23^ and a study in Colorado found a 69% increase in Δ9-tetrahydrocannabinol (THC) concentrations in offspring meconium following legalization of cannabis for recreational use, which is consistent with increases in maternal exposure to THC (eg, via higher use frequency or greater potency).^31^ California legalized recreational cannabis use beginning on January 1, 2018, after a gradual 2-decade expansion of legal medical cannabis markets, and it is unknown whether daily prenatal cannabis use has escalated more rapidly following this policy change. As states continue to legalize cannabis and permissive attitudes toward cannabis increase, continuously monitoring changes in cannabis use frequency during pregnancy will be essential.

### Strengths and Limitations

This study has several key strengths, including the large sample size of sociodemographically diverse pregnant women, universal screening for self-reported use of cannabis in the year before and during pregnancy as part of standard prenatal care, and longitudinal data spanning 9 years. This study, however, also has several limitations. Our study was limited to self-reported cannabis use among women in KPNC who were screened for substance use when they began prenatal care and results may not be generalizable to women without health care access or to women outside of California. The prenatal substance use screening questionnaire assessed self-reported prenatal cannabis use at the first prenatal visit (at approximately 8 weeks’ gestation) and does not reflect continued use throughout pregnancy. In addition, we cannot differentiate whether cannabis use during pregnancy occurred before or after women were aware that they were pregnant. This is important because studies suggest that women report quitting or decreasing frequency of cannabis use when they learn they are pregnant.^32^ Prenatal cannabis use is underestimated by self-report,^7^ and self-reported use was lower than national data,^6,11^ providing additional support that women may not disclose cannabis use in health care settings. Furthermore, women who self-report cannabis use before or during pregnancy may underreport the frequency with which they use, and daily and weekly cannabis use in pregnancy may be underestimated in this study.

## Conclusions

Results of this study show that the frequency with which women in California use cannabis in the year before and during pregnancy has increased over time, corresponding with increasing acceptance of cannabis use and decreasing perceptions of cannabis-associated harms. Future studies are critically needed to determine whether and how the adverse outcomes of maternal perinatal cannabis use on the health and development of infants and children vary with daily vs less frequent use.
